# Role of the MAPK pathway in human lung epithelial-like A549 cells apoptosis induced by paraquat

**DOI:** 10.1590/1678-4685-GMB-2019-0137

**Published:** 2020-03-30

**Authors:** Da-Zhuang Sun, Chun-Qing Song, Yong-Min Xu, Xue-Song Dong

**Affiliations:** 1 Department of Emergency, The First Affiliated Hospital, China Medical University, Shenyang 110001, China.

**Keywords:** Paraquat, A549 cells, MAPKs, mitochondria, apoptosis

## Abstract

This study aims to investigate the value of mitogen-activated protein kinases (MAPKs) for paraquat (PQ)-induced apoptosis in human lung epithelial-like A549 cells and the specific mechanism. A549 cell apoptosis were induced by PQ. These cells were divided into six groups: control group (cells were cultured in RPMI-1640 medium); SP600125 group (cells were preconditioned with SP600125); SB203580 group (cells were preconditioned with SB203580); PQ group (cells were treated with PQ); SP600125+PQ group (cells were preconditioned with SP600125 following PQ); SB203580+PQ group (cells were preconditioned with SB203580 following PQ). The cell survival rate, apoptosis rate, and activities of caspase-3 and -9 were detected. When compared with the control group, both SP600125 and SB203580 groups had no significant difference in the detected indicators. When compared with PQ group, the cells in both SP600125+PQ group and SB203580+PQ group had significantly increased viability and level of anti-apoptotic protein Bcl-2; and had decreased apoptotic rates, decreased levels of caspase-3 and -9, and decreased level of pro-apoptotic protein Bax. The ratio of p-JNK/JNK protein expression in the SP600125+PQ group significantly decreased, while the ratio of the p-P38/P38 protein expression in the SB203580+PQ group decreased. PQ induced A549 cell apoptosis through the MAPKs pathway.

## Introduction

Paraquat (PQ), which is named as 1,1’-dimethyl-4,4’-bipyridinium dimethosulfate, is a highly effective quaternary amine herbicide widely used in the world. Due to its high toxicity and lack of effective treatment measures, it has extremely high poisoning mortality. Most patients with PQ poisoning die from acute lung injury (ALI) or progressive pulmonary fibrosis. The apoptosis of alveolar epithelial cells (AECs) caused by PQ poisoning plays a key role in the development of ALI, and the occurrence of pulmonary interstitial fibrosis in the later stage (Chen *et al.*, 2012; Sun and Chen, 2016). To date, the molecular mechanism of the apoptosis of AECs induced by PQ remains not fully understood. The previous studies of the investigators have revealed that PQ induces the apoptosis of human lung epithelial-like A549 cells by activating the mitochondrial apoptotic pathway (Sun *et al.*, 2017). A recent study revealed that the activation of mitogen-activated protein kinases (MAPKs) may be involved in the molecular mechanism of PQ-induced apoptosis (Pang *et al.*, 2016). MAPKs are ser/Thr kinases that respond to various stresses and regulate cellular responses. The MAPK pathway consists of several parallel signaling pathways. At present, three parallel MAPK pathways have been found in mammalian cells: the ERK pathway, the c-Jun N-terminal kinase (JNK) pathway, and the p38 Mapk pathway (Cargnello *et al.*, 2011). In recent years, more and more studies have revealed that JNK and p38 are activated through phosphorylation under the stimulation of various cell stress factors, induce the expression of apoptotic protein Bax, and inhibit the expression of anti-apoptotic protein Bcl-2, leading to Bax-mediated mitochondrial apoptosis (Kim *et al.*, 2015; NavenKumar *et al.*, 2017; Wang *et al.*, 2018). However, its role in PQ poisoning and its mechanism remain unclear.

Therefore, the investigators conducted this study to investigate the importance of MAPKs for PQ-induced apoptosis in human lung epithelial-like A549 cells and the specific mechanism.

## Materials and Methods

### Materials

Human A549 cells were obtained from the Experimental Center of China Medical University. Fetal bovine serum was purchased from Gemini (USA). The Roswell Park Memorial Institute (RPMI) 1640 medium was purchased from Hyclone (USA). PQ, trypsin, MTT and dimethyl sulfoxide (DMSO) were purchased from Sigma (USA). SP600125, SB203580, ECL luminescent liquid, cell lysate and the bicinchoninic acid (BCA) quantification kit were purchased from Beyotime Biotechnology Research Institute (China). The caspase activity detection kit was purchased from Nanjing Keygen Biotech (China). The annexin V-FITC cell apoptosis detection kit was purchased from DOJINDO (Japan). The JNK, phospho-JNK(p-JNK), p38 and phospho-p38(p-P38) antibodies were purchased from Bioworld Technology (USA).

### Methods

#### Cell cultivation and grouping

Cells were cultured in RPMI-1640 medium containing 10% fetal bovine serum, and placed in an incubator at 37 °C with 5% CO_2_.

Experimental grouping: (1) control group: cells were added with an equal volume of RPMI-1640 medium; (2) SP600125 group: cells were pretreated with medium containing the JNK-specific inhibitor SP600125 (10 μM) for two hours; (3) SB203580 group: cells were pretreated with medium containing p38-specific inhibitor SB203580 (10 μM) for two hours; (4) PQ group: cells were cultured in medium containing PQ (200 μM); (5) SP600125+PQ group: cells were pretreated with medium containing SP600125 (10 μM) for two hours, and subsequently added with medium containing PQ (200 μM); (6) SB203580+PQ group: cells were pretreated with medium containing SB203580 (10 μM) for two hours, and subsequently added with medium containing PQ (200 μM). Cells in each group were treated according to the experimental groups, and the culture was continued for 48 hours.

#### Detection of cell viability

Cells were seeded in a 96-well plate. After cells were treated according to the experimental groups, 20 μL of 5 mg/mL of MTT was added to each well, shaken to mix, and cultured at 37 °C with 5% CO_2_ for 4 h. Then, the supernatant in each well was blotted up, 150 μL of DMSO was added to each well, and this was shaken to sufficiently dissolve the precipitation. The absorbance values were determined using an enzyme micro-plate reader at a wavelength of 570 nm.

#### Detection of cell apoptosis rate

Cells were seeded in a six-well plate. After cells attached to the wall, cells were treated according to the experimental groups. After incubation, two groups of cells were collected with trypsin without ethylenediaminetetraacetic acid (EDTA), centrifuged at 10,000 rpm for 5 min, washed with phosphate buffered saline (PBS) for two times, added with 5 μL of annexin V-FITC and 10 μL of propidium iodide (PI), and incubated in the dark at room temperature for 15 min. The cell apoptosis rate (Leith *et al.*, 2017) was detected by flow cytometry.

#### Detection of caspases activity

After A549 cells were treated according to the experimental groups, cells in all groups were collected, washed with PBS three times, and added with the appropriate amount of lysis buffer. When these cells were completely lysed, these were centrifuged at 10,000 rpm for 1 min. Then, the supernatant was collected, and the BCA protein quantitative kit was used to detect the protein concentration. The protein concentration was set at 3 μg/μL. Then, 50 μL of cell lysis product was drawn, added with 50 μL of 2 buffer and 5 μL of the reaction substrate of caspase, and incubated in the dark at 37 °C for four hours. The absorbance values were determined using an enzyme micro-plate reader at a wavelength of 405 nm.

#### Western blot

Cells in all groups were collected, the protein was extracted, and the protein concentrations in all groups were determined using a BCA kit. Then, the samples were separated by 12% sodium dodecyl sulfate polyacrylamide gel electrophoresis (SDS-PAGE), transferred onto a polyvinylidene fluoride (PVDF) membrane with wet electricity, and the membrane was blocked with 5% dried skimmed milk at room temperature for two hours. Afterwards, this was added with JNK, p-JNK, p38, p-P38, Bax, Bcl-2 and β-actin antibodies diluted by tris-buffered saline and Tween 20 (TBST), and left standing overnight at 4 °C. Next, the membrane was washed, added with the secondary antibody diluted by TBST, placed in room temperature for two hours, colored with ECL luminescent liquid, and developed and fixed. The gray analysis was carried out using the ImageJ image analysis software.

### Statistical analysis

Data were statistically analyzed using statistical software SPSS 20.0. Data in all groups were expressed as mean ± standard deviation. Intergroup comparison was conducted using univariate analysis of variance. When the variance was homogeneous, intergroup pairwise comparison was conducted using Dunnett *t*-test. When the variance was heterogeneous, intergroup pairwise comparison was conducted using Dunnett’s T3-test. *P* < 0.05 was considered statistically significant.

## Results

### Changes in cell viability

**Figure 1:**
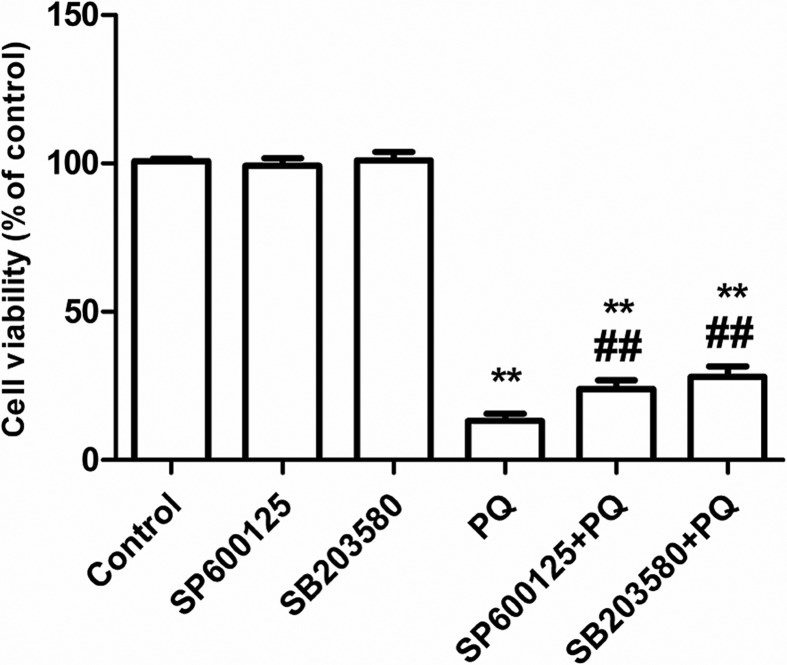
Effect of blocking MAPKs pathway on PQ-induced cytotoxicity. **P < 0.01 vs control group; ^##^ *P* < 0.01 vs PQ alone group.

After A549 cells were treated according to the experimental groups, changes in the activity of A549 cells were analyzed using the tetrazolium salt colorimetric assay (MTT) method. The results revealed that the differences in cell viability between the SP600125 group and control group, as well as between the SB20358 group and control group, were not statistically significant. PQ significantly reduced the viability of A549 cells, and the pretreatment with SP600125 or SB203580 significantly inhibit PQ-induced cell viability reduction (Figure 1). The results revealed that the inhibition of the JNK MAPK or p38 MAPK pathways could reduce the cytotoxicity of PQ.

### Changes in cell apoptosis rate

**Figure 2:**
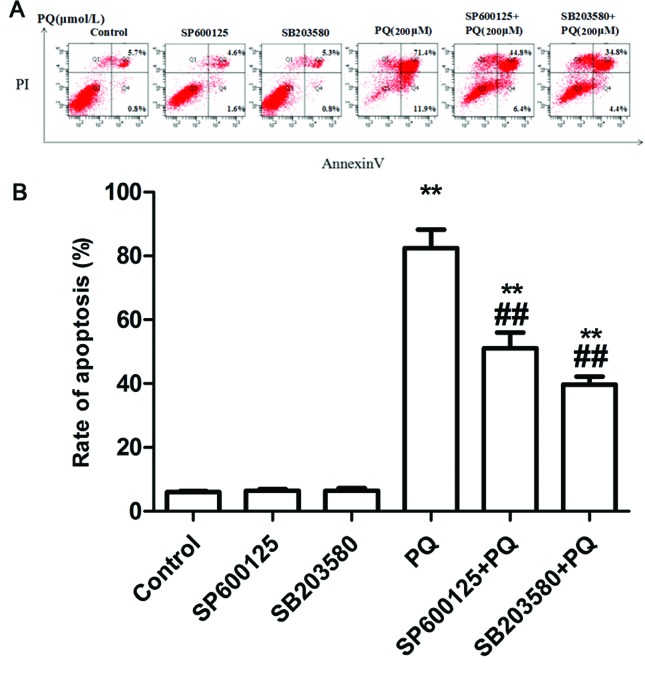
A: Flow cytometry analysis of apoptotic rates in each group; B: Statistical analysis of apoptotic rates in each group. **P < 0.01 vs control group; ##*P* < 0.01 vs PQ alone group.

A549 cells were treated according to the experimental groups, underwent and annexin V-FITC/PI double staining. Changes in apoptosis were detected by flow cytometry. These results suggest that the differences in cell apoptosis rate between the SP600125 group and control group, as well as between the SB20358 group and control group, were not statistically significant. The apoptosis rate of A549 cells was significantly higher in the PQ group than in the control group. Pretreatment with SP600125 or SB203580 could protect cells, and reduce PQ-induced apoptosis (Figure 2).

### Changes in caspase-3/-9 activity

**Figure 3:**
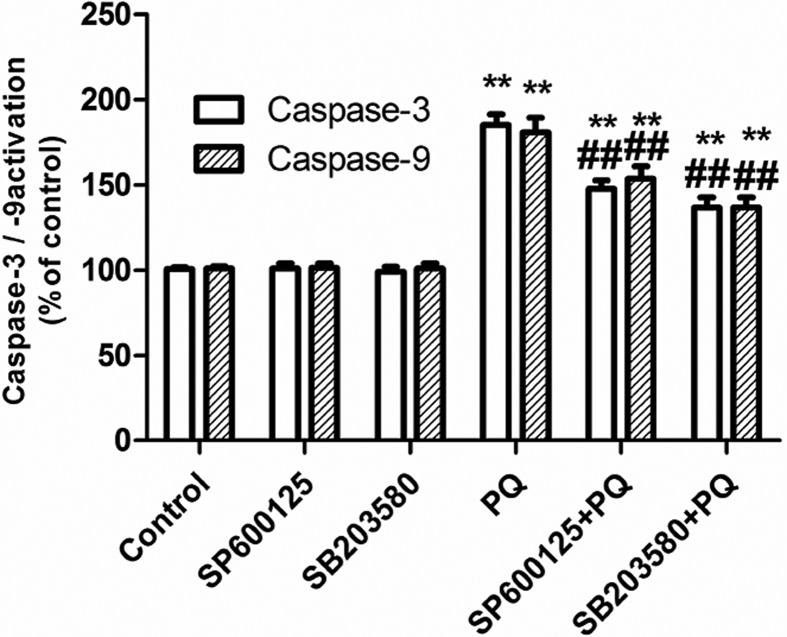
Effect of blocking of MAPKs pathway on the activity of PQ induced Caspase-3/-9. **P < 0.01 vs control group; ##*P* < 0.01 vs PQ alone group.

Endogenous apoptotic pathways do not require cell surface receptors, but rely on participating mitochondria. When cells are stimulated by apoptotic factors, cytochrome C is released from the mitochondria, binds with Apaf1 to form an apoptotic complex, and activates caspase-9. Then, the apoptotic complex activates caspase-9 and activates the downstream caspase-3/-6/-7, in order to mediate mitochondrial apoptosis in cells. These experimental results revealed that the differences in activities of caspase-3 and caspase-9 between the SP600125 group and control group, as well as between the SB20358 group and control group, were not statistically significant. PQ significantly increased the activities of caspase-3 and caspase-9 in A549 cells. However, the pretreatment with SP600125 and SB203580 significantly reduced the PQ-induced increase in activities of caspase-3 and caspase-9 in A549 cells (Figure 3).

### Changes in the protein expression of related MAPKs and mitochondrial apoptotic signaling pathways

**Figure 4:**
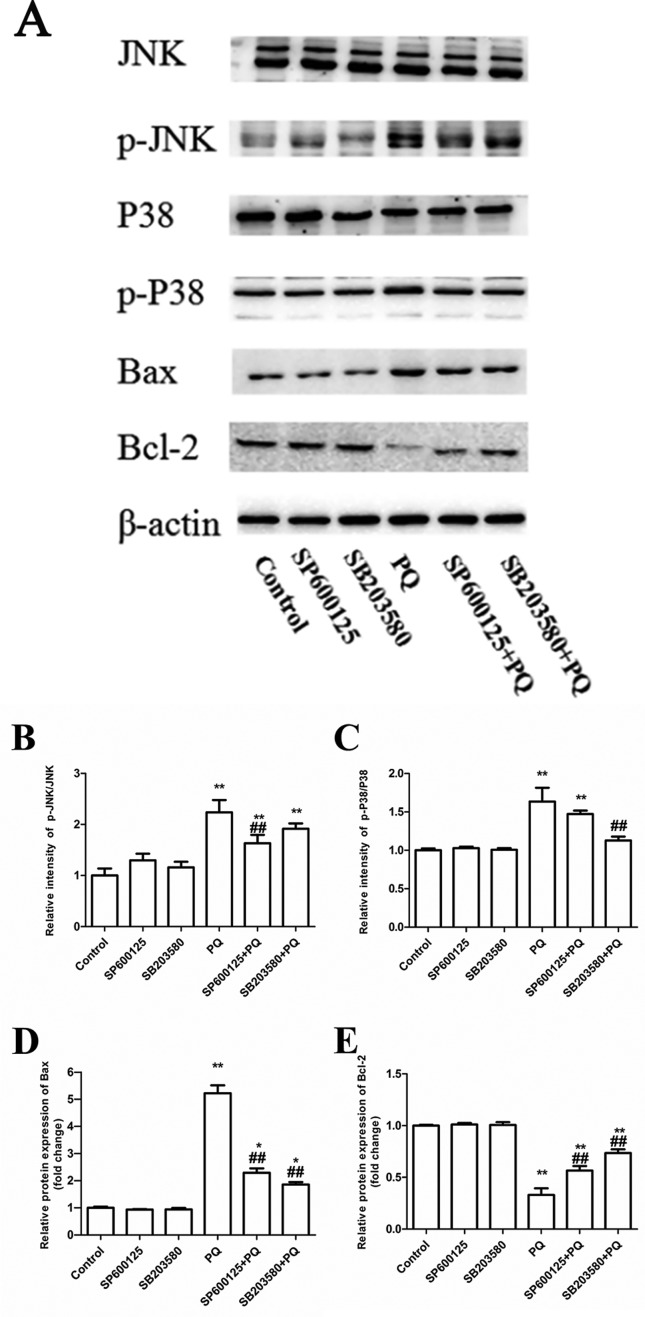
Effect of blocking MAPKs pathway on the expression of related proteins changes of MAPKs and mitochondrial apoptosis signal transduction pathway in PQ-induced apoptosis. A: Western blotting results; B: p-JNK/JNK levels; C, p-P38/P38 levels; D: Bax protein; E: Bcl-2 protein. *P < 0.05, **P < 0.01 vs control group; ##*P* < 0.01 vs PQ alone group.

After A549 cells were treated according to the experimental groups for 48 hours, Western blotting was performed to detect the protein expression of JNK, p-JNK, p38, p-P38, Bax, Bcl-2 and β-actin. These results revealed that the differences in the expression of the above-mentioned proteins between the SP600125 group and control group, as well as between the SB20358 group and control group, were not statistically significant. SP600125 could significantly reduce the proportion of the PQ-induced expression of p-JNK/JNK, decrease the expression of Bax, and increase the expression of Bcl-2 protein in A549 cells. SB203580 could significantly reduce the proportion of the PQ-induced expression of p-P38/P38, decrease the expression of Bax, and increase the expression of Bcl-2 protein in A549 cells (Figure 4).

## Discussion

The apoptosis of AECs plays an important role in the pathophysiological process of PQ toxicity (Chen *et al.*, 2012; Sun and Chen, 2016). The intake of PQ *in vivo* can be transported in the reverse concentration gradient and accumulated into types I and II AECs through the polyamine transport/uptake system. The accumulation of PQ leads to histopathological changes, such as the necrosis and apoptosis of cells, causes significant damage and destruction of alveolar epithelium, and causes gas exchange dysfunction and deficiency of surfactant, leading to clinical manifestations, such as alveolitis, ALI and pulmonary interstitial fibrosis (Dinis-Oliveira *et al.*, 2008). The PQ-induced apoptosis of AECs involves a variety of signaling pathways. Mitochondrial pathway-mediated apoptosis plays an important role. The previous study of the investigators confirmed that PQ induced the apoptosis of human lung epithelial-like A549 cells through the mitochondrial apoptosis pathway, and it was also revealed that p53 protein was an important regulator in the apoptotic process (Sun *et al.*, 2017).

The MAPK signaling pathway plays an important role in regulating apoptosis under stress conditions. Studies have revealed that JNK and p38 MAPK were activated by their own phosphorylation to further mediate the mitochondrial apoptosis pathway under the stimulation of various cytokines (Kim *et al.*, 2015; NavenKumar *et al.*, 2017; Wang *et al.*, 2018). The p-jnk /JNK or p-p38/ p38 level can be used to monitor the activity of JNK and p38 (Pei *et al.*, 2014; Shen *et al.*, 2017; Abhilasha, *et al.*, 2019; Wang *et al.*, 2019). The mitochondrial apoptotic pathway mainly controls the integrity of mitochondria through protein interaction between pro-apoptotic and anti-apoptotic proteins in the Bcl-2 protein family (Adams and Cory, 2007; Llambi and Green, 2011). Normally, Bax exists in the cytoplasm in a soluble form. Once activated by apoptotic stimulants, Bax is transferred from the cytoplasm to mitochondria, leading to changes in mitochondrial permeability, which accordingly reduces the potentials of the mitochondrial membrane (Antonsson *et al.*, 2001). Then, cytochrome C is released from the mitochondria to activate caspase-9 and caspase-3, finally inducing apoptosis.

Mounting evidence in recent years has revealed that PQ-induced apoptosis involves the MAPKs signaling pathway. A present study revealed that PQ prolonged the lifetime of neutrophils by activating the p38 MAPK signaling pathway, inducing the production of reactive oxygen species and increasing the concentration of TNF-α (Wang *et al.*, 2014). Peng *et al.* (2004) revealed that the JNK signaling cascade was a direct activator of the mechanism of the PQ-mediated apoptosis of substantia nigra dopaminergic neurons, and this also provided the molecular correlation between oxidative stress and neuronal apoptosis.

In general, the activation of ERK1/2 promotes cell proliferation and acts against the proapoptotic functions of stress-activated JNK and p38 MAPK pathways (Junttila *et al.*, 2008; Munshi and Ramesh, 2013). Hu *et al.* (2017) revealed that MAPKs were involved in the process of PQ-induced lung injury in SD mice, and played an important role. However, the role of the MAPK signaling pathway in the PQ-induced apoptosis of AECs remains unclear. In the present study, the investigators observed whether PQ-induced apoptosis in A549 cells could be reduced by pre-treating JNK MAPK with the JNK-specific inhibitor (SP600125) and pre-treating p38 MAPK with the p38-specific inhibitor (SB203580). The results revealed that both SP600125 and SB203580 could alleviate the PQ-induced apoptosis of A549 cells, and it was presented that PQ induced the apoptosis of A549 cells through the JNK/P38-mediated mitochondrial apoptotic signaling pathway. However, in our study, the use of a transformed cell line as a model of type II AECs, such as the existence of potential differences regarding the apoptosis activation pathways in a cell line originated from a lung adenocarcinoma compared to normal AECs remains unknown and should be further researched. Besides, activation of JNK and p38 kinases pathways was not the sole molecular event associated to PQ exposure. Other molecular processes associated to PQ cytotoxicity maybe important in human lung epithelial-like A549 cells apoptosis induced by PQ.

This study has certain limitations. Firstly, in this trial, the investigators found that SP600125 and SB203580 inhibited the kinases upstream of JNK and P38, and thus inhibited their phosphorylation. However, it was better to further evaluate the effective inhibition of JNK and P38 kinases by analyzing the downstream substrates of these two kinases. The investigators will further look into this issue in our future study. Secondly, this present study aims to investigate the role of MAPKs and the mechanism during PQ-induced apoptosis in human lung epithelial-like A549 cells. However, there were not enough data about the normal alveolar cell line model. We will further investigate this in our next trial.

## Conclusion

In summary, in the present study, human lung epithelial-like A549 cells were used as a model of human AECs to evaluate the mechanism of PQ-induced apoptosis. These results suggest that PQ induces the apoptosis of A549 cells through the MAPK-mediated mitochondrial apoptotic signaling pathway. This provides experimental and theoretical bases for the research and development of new drugs.
